# A Protocol for a Systematic Review on Septic Arthritis of the Temporomandibular Joint (SATMJ)

**DOI:** 10.3390/jcm14072392

**Published:** 2025-03-31

**Authors:** Karolina Lubecka, Kacper Galant, Maciej Chęciński, Kamila Chęcińska, Filip Bliźniak, Agata Ciosek, Tomasz Gładysz, Katarzyna Cholewa-Kowalska, Dariusz Chlubek, Maciej Sikora

**Affiliations:** 1 Department of Oral Surgery, Preventive Medicine Center, Komorowskiego 12, 30-106 Kraków, Poland; lubeckarolina@gmail.com (K.L.); fblizniak@gmail.com (F.B.); 2 Faculty of Medicine, Medical University of Lodz, Al. Kościuszki 4, 90-419 Lodz, Poland; kacpergalant.ld@gmail.com (K.G.); agata.ciosek@stud.umed.lodz.pl (A.C.); 3 National Medical Institute of the Ministry of Interior and Administration, Wołoska 137, 02-507 Warsaw, Poland; maciej@checinscy.pl (M.C.); checinska@agh.edu.pl (K.C.); sikora-maciej@wp.pl (M.S.); 4 Department of Maxillofacial Surgery, Hospital of the Ministry of Interior, Wojska Polskiego 51, 25-375 Kielce, Poland; 5 Department of Oral Surgery, Medical College, Jagiellonian University, Montelupich 4, 31-155 Kraków, Poland; t.gladysz@uj.edu.pl; 6 Department of Glass Technology and Amorphous Coatings, Faculty of Materials Science and Ceramics, AGH University of Krakow, Mickiewicza 30, 30-059 Kraków, Poland; cholewa@agh.edu.pl; 7 Department of Biochemistry and Medical Chemistry, Pomeranian Medical University, Powstańców Wielkopolskich 72, 70-111 Szczecin, Poland

**Keywords:** septic arthritis, temporomandibular joint, temporomandibular disorders, protocol, systematic review

## Abstract

**Background/Objectives:** Septic arthritis of the temporomandibular joint is an infectious disease with a rapid course and possible long-term complications. It is crucial to diagnose and implement treatment quickly and to know the potential causes of the occurrence of SATMJ. The planned systematic review aims to summarize current knowledge on this subject. **Methods:** This protocol follows the Preferred Reporting Items for Systematic Reviews and Meta-Analyses (PRISMA) guidelines and checklist. The following scientific databases will be searched: ACM, BASE, ClinicalTrials.gov, Cochrane Library, Google Scholar, PubMed, and Scopus. Studies on SATMJ that are consistent with the pre-established PICOTS criteria will be included in the systematic review. Two authors will independently conduct the record screening, data extraction, and quality appraisal phases. The quality of the studies will be evaluated using the JBI critical appraisal tools. Certainty assessment will be conducted using the GRADE tool. The obtained research results and data will be used to define and establish the current scientific position on the diagnosis and treatment of SATMJ. Conclusions on the lack of association of gender, age, and race with the occurrence of this disease entity will be verified, among others. The planned systematic review will be based on extensive searches for studies with no high risk of bias. The aim is to assist clinicians in managing SATMJ, and to inspire future research.

## 1. Introduction

The temporomandibular joint (TMJ) is a paired structure located anteriorly and inferiorly to the external auditory canals [1,2]. Anatomically, it consists of a glenoid fossa on the temporal bone and a head on the condylar process of the mandible [1,2,3]. The surfaces of these bones are covered with cartilage and separated by a cartilaginous joint disc [1,2,3,4]. The disc divides the joint cavity into superior and inferior compartments [3]. The surfaces mentioned above, along with the joint capsule, determine the boundaries of the joint cavity [5]. It is filled with synovial fluid, the main component of which is hyaluronic acid [6]. It also contains substances such as lubricin and enzymes: proteinases and collagenases [7,8]. Its basic functions are nourishing the cartilage, absorbing shock, transferring loads, decreasing friction, and lubricating the joint [2,9,10]. Among the most common problems with the TMJ are temporomandibular disorders (TMDs), concerning approximately 31–34% of adults and 11% of adolescents, according to the newest knowledge [11,12,13]. There are a lot of methods for treating TMDs, including surgical ones, such as administering non-steroidal anti-inflammatory drugs or hyaluronic acid mixtures into the joint cavity [4,14]. TMJ infections are much rarer than TMDs, with fewer than 100 cases reported in the literature to date [15]. However, hospitals around the world must deal with these conditions without proper protocols, due to the rarity of these diseases.

Infection of the TMJ cavity is called septic arthritis of the TMJ (SATMJ). This condition occurs so rarely that there is a lack of clear principles for its diagnosis and treatment in the specialist literature [16]. At the same time, SATMJ is so common that every maxillofacial surgeon can encounter it. As an acute condition, it requires immediate and precise treatment [16,17]. The anatomical richness of the area hiding the TMJ contributes not only to the diverse etiology of SATMJ but also to the risk of numerous complications of untreated or poorly treated inflammation [16,17]. These include irreversible damage to the structures of the TMJ itself, the external auditory canal, and the valuable facial nerve [16,17]. Turton et al. even described the spread of infection to the middle cranial fossa as a consequence of SATMJ. Therefore, it is important to develop clinicians’ awareness of this disease entity and increase their vigilance regarding the diagnosis. The occurrence of nonspecific symptoms constituting the cardinal features of inflammation, i.e., *rubor*, *calor*, *tumor*, *dolor*, and *functio laesa* (redness, heat, swelling, pain, and loss of function), in combination with *trismus* and *malocclusio* (lockjaw and malocclusion), should prompt the doctor to consider this infection [18,19].

Due to the low incidence of SATMJ, as mentioned above, but also the high probability of this disease entity being encountered by every maxillofacial surgeon in their medical practice, it is important and clinically necessary to summarize the current research and observations on this topic in a single, scientifically correct systematic review [20]. This could provide an explanation of the problem and, at the same time, a ready-made method of treatment for clinicians dealing with SATMJ, thus saving time and money for hospitals that accept such patients [21].

It should also be remembered that SATMJ is an acute disease with a rapid course that requires quick and effective treatment. Its neglect can lead to serious health consequences, as there are documented cases of death due to SATMJ. It is always good to prepare well in advance for how to deal with such diseases logistically and knowledge-wise [22]. Despite the lack of standardization in SATMJ treatment costs, they can range from a few hundred USD to hundreds of thousands of USD [23]. They can therefore be a significant financial burden for patients.

Three teams of authors have undertaken systematic reviews of the literature on SATMJ to date: Gera et al., Jovanović et al., and Omiunu et al. [18,19,24]. The authors’ teams identified 25, 37, and 38 papers describing SATMJ cases, respectively [18,19,24]. However, only seven of the identified reports overlap in all three works [18,19,24].

Identified cases were synthesized in a readable manner; hence, none of the systematic reviews rejected cases whose presentations were characterized by a significant risk of bias [18,19,24]. Syntheses were conducted without distinguishing subgroups, partially precluding their practical applicability [18,19,24]. This does not in any way diminish the work of the research teams discussed. However, an average clinician expects more precise answers to the issues of proper diagnostic and therapeutic procedures. Clear connections are expected among risk factors, clinical presentation, test results, and the therapeutic path choice. Currently, there are several popular approaches, and it is impossible to speak of a universal set of procedures [19].

Therefore, despite the existence of systematic reviews with syntheses conducted, a need for further exploration of the subject of SATMJ can be seen. The literature will be searched again, more extensively, including references from the previous reviews and cases newer than those included in the discussed reviews. Reports with a high risk of bias are to be excluded. A qualitative and quantitative synthesis is planned.

The rarity of SATMJ leads to even leading maxillofacial surgery centers referring patients to other hospitals to be treated by clinicians who have already encountered the condition [21]. Summarizing information about SATMJ would streamline the chain of care for these patients, providing them with appropriate care right from the start. This would provide a double benefit—patients would recover faster, and hospitals would operate more efficiently. Especially in the light of evidence-based medicine, it is important to classify and summarize all scientific achievements on an ongoing basis, especially when they concern diseases that have a real impact on the health and well-being of the population [25].

This review will aim to address two main issues, primarily, (1) to evaluate the effectiveness of surgical combined with conservative treatment versus conservative treatment alone in managing SATMJ and, secondarily, (2) to identify the most common determinants, including demographic and health-related characteristics of affected patients.

The first research question will explore whether surgical treatment combined with conservative treatment is more effective in the therapy of SATMJ than conservative treatment alone. For this purpose, there will be a comparison between the types of therapies used in terms of their impact on combating inflammatory symptoms and parameters, TMJ function, and long-term follow-ups.

The article is also intended to determine the most common risk factors and etiological factors responsible for the occurrence of SATMJ. The age, sex, and origin of the patients who suffer from it most frequently will be analyzed. A particular emphasis will be placed on the health status: both systemic and local diseases, as well as TMJ area injuries, will be taken into account. In addition, the routes of spread of infection and the pathogens identified in the TMJ cavity will be analyzed.

Finally, there will be a search for connections between the variables mentioned, through qualitative and quantitative analyses. Among the latter, the greatest potential is seen in correlation and regression analyses.

The following hypotheses will be verified:

**H₀-1.**  *The incidence of septic arthritis of the temporomandibular joint (SATMJ) is not associated with the patient’s sex, age, race, comorbidities, immunological status, or overall health condition*.

**H₀-2.**  *There is no predominance of any particular route of infection spreading to the temporomandibular joint (TMJ), including otogenic, odontogenic, hematogenous, or other pathways*.

**H₀-3.**  *None of the available diagnostic methods for SATMJ (anamnesis, physical examination, imaging studies, laboratory tests, microbiological examination, or histopathological analysis) demonstrate superior sensitivity, specificity, accuracy, or precision compared to the others*.

**H₀-4.**  *There are no significant differences in treatment outcomes, length of hospitalization, or recurrence rates between conservative, invasive, and combined conservative–invasive approaches for SATMJ*.

## 2. Methods

The planned systematic review was pre-registered on November 23, 2024, in the PROSPERO International prospective register of systematic reviews (Centre for Reviews and Dissemination, University of York, York, UK), under number CRD42024613462. The workflow for the SATMJ publication series is as follows: preliminary searches (performed in autumn 2024); overview of systematic reviews (published in January 2025); protocol for the new, most extensive and thorough systematic review to date (this article); proper systematic review. The PROSPERO submission contains the basic elements of the protocol that is presented in detail in this chapter.

The protocol was developed under the Preferred Reporting Items for Systematic Reviews and Meta-Analyses (PRISMA), 2020 version [26]. The following subsections reflect the Methods section of the 2020 PRISMA checklist, an Appendix A to the guidelines [26].

### 2.1. Eligibility Criteria

Due to the superiority of PICO(T)(S) over SPIDER for systematic reviews that are to be completed with a quantitative synthesis and meta-analysis, the first method was chosen [5,27]. The acronym was expanded as follows: P—patients, understood as the study sample; I—intervention, understood as the entire diagnostic and therapeutic procedure; C—comparison, understood as a control sample; O—outcomes, understood as qualitative or quantitative results of the diagnostic and therapeutic process; T—timeframe, understood as the range of publication dates of reports included in the review; S—settings, understood as technical characteristics of the reports [27].

It was planned to include patients with TMJ inflammation diagnosed by medical history and physical examination. Due to the high specificity of this disease entity, it was determined that relying on the subjective assessment of the clinician would be sufficient [28]. Therefore, it will not be required to present the result of the microbiological test for the inclusion of the report in the review. This is particularly important for cases where microorganisms from the joint cavity have not been cultured. It is not an exceptional situation and may result from several factors. These include technical errors during swab collection, poorly selected transport medium, transport problems (especially its delay), errors in transfer to the growth medium, and irregularities during incubation [29].

It was decided to exclude cases of SATMJ observed in animals and studies on human cadavers. Due to the anatomical and functional differences and the intention to synthesize and analyze the results, there is a plan to exclude pediatric patients [30].

Any information on the determinants of SATMJ is desirable. The existence of at least rudimentary information on potential risk factors or etiological factors and the routes of spread of SATMJ will be sufficient for the report to be included in the synthesis on determinants.

Any type of medical intervention is to be allowed, as long as it is aimed at treating TMJ disease. Therefore, reports describing local procedures will be included, as well as systemic pharmacotherapy due to SATMJ. The exception will be the treatment of a systemic disease only, even if it could indirectly affect the resolution of SATMJ. For example, treatment of diabetes or sepsis without any specific focus on TMJ is not to be permitted.

Since many of the known SATMJ episodes are described in single-case reports, the requirement for comparison has been waived [1,20,22,28,31]. If a series of cases diagnosed or treated differently are identified, they will be treated individually whenever possible.

No timeframe limitations are planned. Only primary studies will be included. These will probably be case reports and case series, but other types of retrospective and prospective studies will be also allowed, as long as they are based on eligible patients. It is planned to exclude papers that have not undergone the full editorial process (e.g., preprints), as well as conference proceedings and fragments of textbooks.

The eligibility criteria are presented in a user-friendly manner in Table 1.

### 2.2. Information Sources

The following databases will be included: Association for Computing Machinery: Guide to Computing Literature (ACM), Bielefeld Academic Search Engine (BASE), Cochrane Central Register of Controlled Trials (CENTRAL), Elsevier Scopus (Scopus), National Library of Medicine: ClinicalTrials.gov (CT), National Library of Medicine: PubMed, and Embase (Table 2). Gray literature will be searched using Google Scholar. To improve quality and reliability, no language or time restrictions are to be introduced during the research. These databases cover a broad field of science, provide a comprehensive insight into the topic, and contain peer-reviewed papers.

### 2.3. Search Strategy

Based on preliminary searches including the terms “temporomandibular”, “septic”, and “arthritis”, along with the already established eligibility criteria, a detailed query was developed. It was agreed that the priority was to identify as many reports of primary studies as possible, at the expense of increased work for the selectors. In this spirit, the query was modified to be as specific as possible while maintaining satisfactory sensitivity. In the next stage, its contents were optimized to avoid two-word keywords (which could be misinterpreted by the engines) and to be limited to the logical operators “AND” and “OR”, of which there is no doubt about the interpretation. Finally, the query took the form of “(temporomandibular OR temporomandibularis OR TMJ) AND (joint OR articulatio) AND (arthritis OR inflammation OR inflammatory OR infection OR infections OR empyema OR abscess OR osteomyelitis) AND (septic OR suppurative OR pyogenic OR microbial OR bacterial OR gram-positive OR staphylococcal OR fungal OR viral)”.

### 2.4. Selection Process

**Figure 1:**
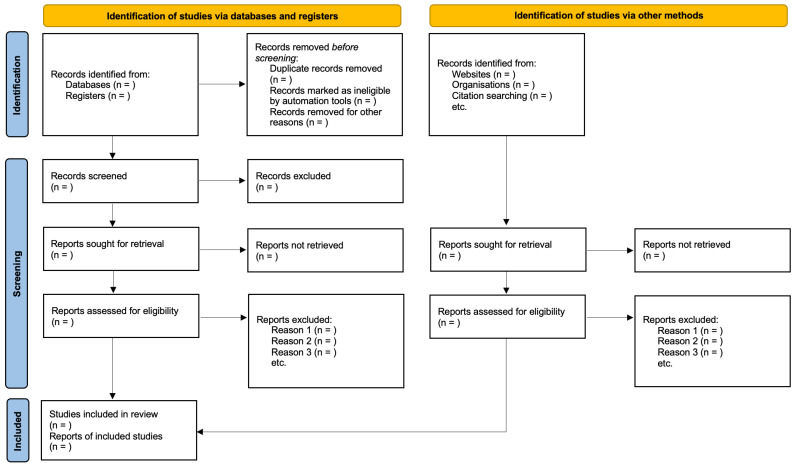
PRISMA 2020 flow diagram template (to be filled).

The records identified in the databases will be entered into the Rayyan automation tool. Then, a manual deduplication will be performed, followed by screening of records based on titles and abstracts by two independent researchers (K.L. and K.G.) in a blinded session. In case of discrepancies in the assessment, the papers will proceed to the next stage. The Cohen’s kappa value will be calculated, expressing the degree of agreement between the researchers. Then, the full-text assessment of the articles will be performed, and in case of disagreement between the researchers, a third researcher (M.C.) will be involved, who will verify (without knowing the decisions of the first two researchers), and a secret vote will be held among the three researchers, the results of which will be binding. To summarize this part of the work and achieve maximum readability of the manuscript, the supplemented PRISMA 2020 flow diagram will be used (Figure 1).

### 2.5. Data Collection Process

Data will be collected independently by two researchers (K.L. and K.G.). In case of any discrepancies between them or divergences in their assessments, a third researcher will be involved (M.C.), whose vote will be the deciding one. Data will be tabulated using the Google Workspace package (Google LLC, Mountain View, CA, USA).

### 2.6. Data Items

For the purpose of identifying determinants, the following data items will be collected, synthesized, and analyzed: (1) demographic data (age, sex), (2) systemic diseases, (3) local injuries or diseases, (4) microbiological agents, and (5) infection spread routes. While all but the latter two are typically treated in systematic reviews as data characterizing patients or groups of patients, the intention here is to consider them as outcomes of medical history, physical examination, and additional tests (mainly laboratory and imaging). Together with microbiological identification of the etiological agent, they form an overall picture of the group experiencing SATMJ. The availability of the discussed data in a systematic form will allow for correlation analysis and further specification of subgroups. This will make it possible to verify whether, for example, skin trauma statistically increases the risk of infection with skin bacteria, or whether complicated odontogenic inflammation is more likely to spread to TMJ in a particular age group.

Additionally, it is planned to collect details on (1) the diagnostic methods used, (2) the implemented conservative treatment (mainly pharmacotherapy), (3) the invasive (surgical) procedures performed, and (4) the occurrence of complications. The data and details to be extracted are presented below in Table 3.

The search for data will focus on the items listed in the table as inclusion criteria. The eligible articles will be scanned to obtain as many data as possible, especially numerical data. In the event that the authors of the studies used different scales or reference points, a unified numerical scale will be used to compare the individual data in the most transparent and scientifically correct way.

### 2.7. Study Risk-of-Bias Assessment

Two independent authors (K.L. and K.G.) will assess the risk of bias using dedicated Joanna Briggs Institute (JBI) critical appraisal tools [32]. These will include, in particular, tools for assessing case series and single case reports. In the event of a discrepancy, a third researcher (M.C.) will also participate in this stage to make an assessment, and a secret vote will be held among the three, the results of which will be binding.

For the case series, there will be 10 dichotomous questions:

1. Are there clear criteria for inclusion in the case series?

2. Was the condition measured in a standard, reliable way for all participants included in the case series?

3. Were valid methods used for identifying the condition for all participants included in the case series?

4. Did the case series have consecutive inclusion of participants?

5. Did the case series have a complete inclusion of participants?

6. Was there clear reporting of the demographics of the participants included in the study?

7. Was there clear reporting of clinical information of the participants?

8. Were the outcomes or follow-up results of cases clearly reported?

9. Was there clear reporting of the presenting sites’/clinics’ demographic information?

10. Was the statistical analysis appropriate?

Case reports will be assessed using 8 yes/no questions:

1. Were the patient’s demographic characteristics clearly described?

2. Was the patient’s history clearly described and presented as a timeline?

3. Was the current clinical condition of the patient on presentation clearly described?

4. Were diagnostic tests or assessment methods and their results clearly described?

5. Were the intervention(s) or treatment procedure(s) clearly described?

6. Was the post-intervention clinical condition clearly described?

7. Were adverse events (harms) or unanticipated events identified and described?

8. Does the case report provide takeaway lessons?

A summary of the number of questions answered affirmatively will be created for each report. In addition, tables will also be created with answers to each of the above questions for each report. A quantitative assessment on a scale of X/10 and X/8 will be made. Only those studies that receive at least 6/10 and 5/8, i.e., a predominance of affirmations, will be included.

### 2.8. Effect Measures

The treatment effects will be measured by numerical values that can be obtained from the research material, such as (1) the number of affected TMJs, (2) types and numbers of different invasive treatments, (3) the number of days of hospitalization, (4) whether there was an inflammation resolution or not (dichotomous meter), and (5) the number of relapses. In addition, the completion and results of the treatment will be also summarized by tabulating the obtained data. MedCalc Software (MedCalc Software Ltd., Ostend, Belgium) and Google Workspace (Google LLC, Mountain View, CA, USA) will be used for this purpose.

### 2.9. Synthesis Methods

Statistical analyses will be performed for determinants, treatment methods, and outcomes. These will be (1) measures of central tendency (mean and median—applicable to all data), (2) measures of dispersion (range and standard deviation—applicable to all data), (3) measures of frequency (percentages—applicable to etiological factors and treatment methods; frequencies—applicable to treatment outcomes), (4) analyses of dependencies and relationships (correlation—applicable to relationships between etiological factors and treatment methods or outcomes; regression—applicable to dependencies between demographic data and outcomes, e.g., age and hospitalization time), and (5) graphical presentation of data (histograms and scatterplots, with trend lines where relevant—applicable to all data for which correlations will be determined or regression models will be attempted).

Based on the reviews so far, it can be expected that the central tendency, measure of dispersion, and measure of the frequency of popularly presented variables will be possible to determine. Pearson’s and Spearman’s correlations will be sought between variables (fitted logistic, linear, and polynomial regression models), and statistical tests will be calculated depending on the type and distribution. There is a plan to present the distributions and fitted regression models in charts. The significance level assumed will be *α* = 0.05. MedCalc Software (MedCalc Software Ltd., Ostend, Belgium) and Google Workspace (Google LLC, Mountain View, CA, USA) will be used for this purpose.

### 2.10. Certainty Assessment

Certainty assessment will be conducted with the use of the Grading of Recommendations Assessment, Development, and Evaluation (GRADE) tool [33]. This is a systematic approach used to evaluate the quality of evidence and the strength of recommendations in healthcare. It assesses the reliability of evidence by considering factors like study design, risk of bias, consistency of results, precision, and applicability. GRADE categorizes evidence into four levels of quality: (1) high—very confident in the effect estimate, (2) moderate—the true effect may differ, (3) low—the true effect is likely to differ significantly, and (4) very low—the true effect is unknown. It also evaluates the strength of recommendations, classifying them as either strong (based on clear benefits outweighing harms, or vice versa) or weak (benefits and harms are closely balanced or uncertain). This framework helps to provide transparent and evidence-based guidance for clinical decision-making [33].

## 3. Discussion

As presented above, it is reasonable to perform a new systematic review on the subject of SATMJ. The reviews that have been created so far do not provide a clear answer to the hypotheses presented in the Introduction above. There is currently no scientific work that would constitute a kind of guide for clinicians dealing with SATMJ and would summarize the latest achievements and observations on this topic.

SATMJ is a disease entity that poses a real challenge, even for experienced clinicians. Its rare occurrence and often rapid course provide the basis for developing a correct protocol for rapid diagnosis and accurate treatment [28]. Currently, there is no clear position of the scientific community on this topic. This often puts doctors in difficult situations. The treatment of SATMJ is not unified, nor are its costs [23]. It is therefore worth delving deeper into this topic in order to provide patients with holistic and modern care worthy of the 21st century [21].

Despite the existence of systematic reviews on SATMJ, a new one is necessary due to the low overlap and the lack of exclusions from the syntheses of reports with a high risk of bias. In this paper, a protocol for a more complete and restrictive systematic review than the previous ones is presented. The proposed systematic review has a high potential for clinical utility because it is planned to show the relationships among risk factors, etiology, manifestations of SATMJ, and the effectiveness of individual therapeutic approaches.

## Figures and Tables

**Table 1:** Eligibility criteria.

	Criteria for Inclusion	Criteria for Exclusion
Patients	SATMJ cases	Animal studies, cadaver studies, pediatric patients
Intervention	Any conservative or surgical treatment	Treatment of a systemic disease only
Comparison	Not required	Not applicable
Outcomes—determinants	Demographics, risk factors, microbiological agents, or spread routes	Not applicable
Outcomes—therapy	Diagnostic methods used, conservative treatment, invasive treatment, hospitalization length, resolution and recurrences, complications	Not applicable
Timeframe	No publication date limit	Preprints
Settings	Primary studies, e.g., case series, case reports	Conference proceedings, book chapters

**Table 2:** Search engine scopes.

Engine	Scope, Records
ACM	3,815,653
BASE	411,948,582
CENTRAL	over 2,000,000
CT	521,206
PubMed	over 37,000,000
Scopus	over 94,000,000
Embase	over 40,000,000

**Table 3:** Data and details to be extracted.

Data	Details
Demographic data (age, sex)	Diagnostic methods used
Systemic diseases	Implemented conservative treatment (mainly pharmacotherapy)
Local injuries or diseases	Invasive (surgical) procedures performed
Microbiological agents	Occurrence of complications
Infection spread routes	

## Data Availability

The original contributions presented in this study are included in the article. Further inquiries can be directed to the corresponding author.

## Appendix A

**Table A1:** Search strategy.

Engine	Query
ACM	[[All: temporomandibular] OR [All: temporomandibularis] OR [All: tmj]] AND [[All: joint] OR [All: articulatio]] AND [[All: arthritis] OR [All: inflammation] OR [All: inflammatory] OR [All: infection] OR [All: infections] OR [All: empyema] OR [All: abscess] OR [All: osteomyelitis]] AND [[All: septic] OR [All: suppurative] OR [All: pyogenic] OR [All: microbial] OR [All: bacterial] OR [All: gram-positive] OR [All: staphylococcal] OR [All: fungal] OR [All: viral]] AND [[All: temporomandibular] OR [All: temporomandibularis] OR [All: tmj]] AND [[All: joint] OR [All: articulatio]] AND [[All: arthritis] OR [All: inflammation] OR [All: inflammatory] OR [All: infection] OR [All: infections] OR [All: empyema] OR [All: abscess] OR [All: osteomyelitis]] AND [[All: septic] OR [All: suppurative] OR [All: pyogenic] OR [All: microbial] OR [All: bacterial] OR [All: gram-positive] OR [All: staphylococcal] OR [All: fungal] OR [All: viral]]
BASE	“(temporomandibular OR temporomandibularis OR TMJ) AND (joint OR articulatio) AND (arthritis OR inflammation OR inflammatory OR infection OR infections OR empyema OR abscess OR osteomyelitis) AND (septic OR suppurative OR pyogenic OR microbial OR bacterial OR gram-positive OR staphylococcal OR fungal OR viral)”
CENTRAL	“(temporomandibular OR temporomandibularis OR TMJ) AND (joint OR articulatio) AND (arthritis OR inflammation OR inflammatory OR infection OR infections OR empyema OR abscess OR osteomyelitis) AND (septic OR suppurative OR pyogenic OR microbial OR bacterial OR gram-positive OR staphylococcal OR fungal OR viral)”
CT	“(temporomandibular OR temporomandibularis OR TMJ) AND (joint OR articulatio) AND (arthritis OR inflammation OR inflammatory OR infection OR infections OR empyema OR abscess OR osteomyelitis) AND (septic OR suppurative OR pyogenic OR microbial OR bacterial OR gram-positive OR staphylococcal OR fungal OR viral)”
PubMed	“(temporomandibular OR temporomandibularis OR TMJ) AND (joint OR articulatio) AND (arthritis OR inflammation OR inflammatory OR infection OR infections OR empyema OR abscess OR osteomyelitis) AND (septic OR suppurative OR pyogenic OR microbial OR bacterial OR gram-positive OR staphylococcal OR fungal OR viral)”
Scopus	“(temporomandibular OR temporomandibularis OR TMJ) AND (joint OR articulatio) AND (arthritis OR inflammation OR inflammatory OR infection OR infections OR empyema OR abscess OR osteomyelitis) AND (septic OR suppurative OR pyogenic OR microbial OR bacterial OR gram-positive OR staphylococcal OR fungal OR viral)”
Embase	“(temporomandibular OR temporomandibularis OR TMJ) AND (joint OR articulatio) AND (arthritis OR inflammation OR inflammatory OR infection OR infections OR empyema OR abscess OR osteomyelitis) AND (septic OR suppurative OR pyogenic OR microbial OR bacterial OR gram-positive OR staphylococcal OR fungal OR viral)”
